# Biomimetic and Biological Nanoarchitectonics

**DOI:** 10.3390/ijms23073577

**Published:** 2022-03-25

**Authors:** Katsuhiko Ariga

**Affiliations:** 1 International Center for Materials Nanoarchitectonics (WPI-MANA), National Institute for Materials Science (NIMS), 1-1 Namiki, Tsukuba 305-0044, Japan; ariga.katsuhiko@nims.go.jp; 2 Department of Advanced Materials Science, Graduate School of Frontier Sciences, The University of Tokyo, 5-1-5 Kashiwanoha, Chiba 277-8561, Japan

**Keywords:** biomimetic structure, biomolecule, enzyme, nanoarchitectonics, nanostructure, nanotechnology, nucleic acid, nucleic acid, protein, self-assembly

## Abstract

A post-nanotechnology concept has been assigned to an emerging concept, nanoarchitectonics. Nanoarchitectonics aims to establish a discipline in which functional materials are fabricated from nano-scale components such as atoms, molecules, and nanomaterials using various techniques. Nanoarchitectonics opens ways to form a more unified paradigm by integrating nanotechnology with organic chemistry, supramolecular chemistry, material chemistry, microfabrication technology, and biotechnology. On the other hand, biological systems consist of rational organization of constituent molecules. Their structures have highly asymmetric and hierarchical features that allow for chained functional coordination, signal amplification, and vector-like energy and signal flow. The process of nanoarchitectonics is based on the premise of combining several different processes, which makes it easier to obtain a hierarchical structure. Therefore, nanoarchitectonics is a more suitable methodology for creating highly functional systems based on structural asymmetry and hierarchy like biosystems. The creation of functional materials by nanoarchitectonics is somewhat similar to the creation of functional systems in biological systems. It can be said that the goal of nanoarchitectonics is to create highly functional systems similar to those found in biological systems. This review article summarizes the synthesis of biomimetic and biological molecules and their functional structure formation from various viewpoints, from the molecular level to the cellular level. Several recent examples are arranged and categorized to illustrate such a trend with sections of (i) synthetic nanoarchitectonics for bio-related units, (ii) self-assembly nanoarchitectonics with bio-related units, (iii) nanoarchitectonics with nucleic acids, (iv) nanoarchitectonics with peptides, (v) nanoarchitectonics with proteins, and (vi) bio-related nanoarchitectonics in conjugation with materials.

## 1. Introduction

In the natural world, highly functionalized systems of living creatures have been developed over billions of years of evolutional processes. The structures of biological systems are rationally organized to express sophisticated functions with high efficiencies and specificities. These elegant organizations are not based on unknown mysteries and can be explained with certainty by scientific principles in physics, chemistry, and biology. However, component variety in biological systems far exceeds that of the components used in conventional artificial systems. In many biological systems, a huge variety of molecules and materials have appropriate structures, are rationally organized, and are functioned in a coordinated and comprehensive manner [1,2,3]. In this sense, biological systems may be said to be the ultimate functional systems that we are aiming for. Based on these backgrounds, the fields related to biomimetic science have been studied for a long time [4,5,6]. These fields have been studied from the viewpoints of general chemistry and biological chemistry, but they need to be reconsidered and updated according to the later developing fields of nano-related science and technology.

**Figure 1:**
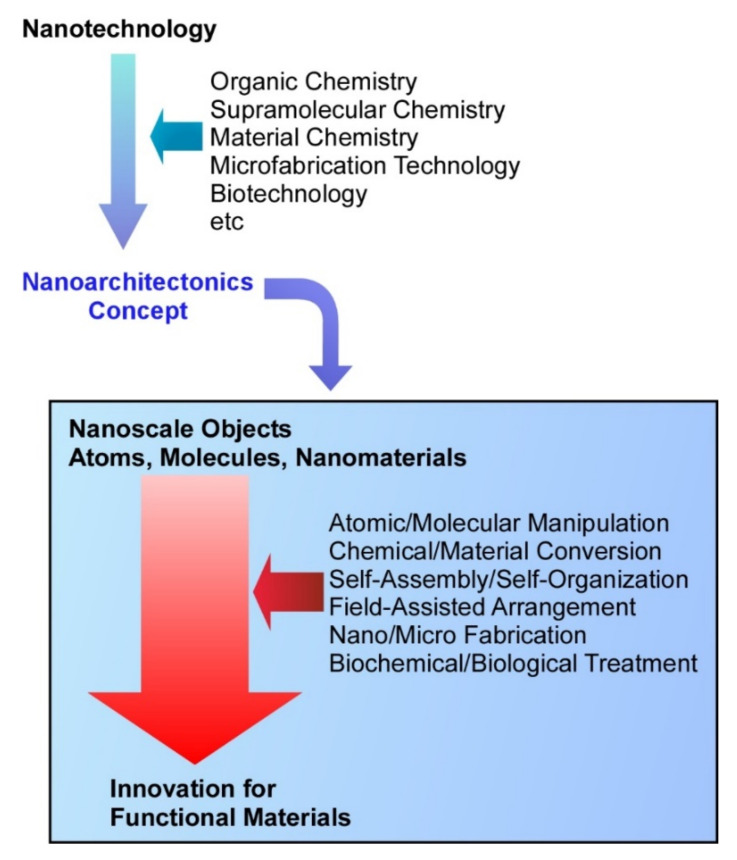
A post-nanotechnology concept, nanoarchitectonics, aiming to fabricate functional materials from nano-scale components such as atoms, molecules, and nanomaterials through the combination of nanotechnology with other research fields, including organic chemistry, supramolecular chemistry, material chemistry, microfabrication technology, and biotechnology.

In general, the creation of functional materials has been pursued using the more orthodox methodologies of organic chemistry [7,8,9], inorganic chemistry [10,11,12], supramolecular chemistry [13,14,15], coordination chemistry [16,17,18], polymer chemistry [19,20,21], and other material sciences [22,23]. It has gradually become clear that functionality depends not only on the properties of the substance itself, but also on how its structure is controlled and how its components are arranged. In parallel, nanotechnology has been rapidly developed. The development of observation and manipulation techniques at the nanoscale, which extends to the molecular and even atomic levels, has led to the discovery and understanding of nanoscale phenomena [24,25,26,27]. In addition, these scientific and technological advances have promoted understanding and advancements in related fields. The development of microfabrication techniques has also stimulated the advancement of device technologies [28,29,30]. In supramolecular chemistry, research on specific molecular recognition [31,32,33] and self-assembly [34,35,36] has been actively pursued. In biological chemistry, the importance of nanostructures in functional systems has also been revealed with the advancement of analytical technology [37,38]. To promote these parallel developments in science and technology in a more rational manner, they should be treated as a unified concept. In particular, we should emphasize the fact that nanotechnology has played the role of a game changer in the observation and elucidation of nanostructures [39,40]. In order to harness these developments for the production of functional materials, a post-nanotechnology concept is necessary. This role has been assigned to an emerging concept, nanoarchitectonics (Figure 1) [41].

Just as Richard Feynman paved the way for nanotechnology [42,43], the concept of nanoarchitectonics was proposed by Masakazu Aono [44,45]. Nanoarchitectonics aims to establish a discipline in which functional materials are fabricated from nano-scale components such as atoms, molecules, and nanomaterials using various techniques [46]. This idea has been actually considered in many fields. Therefore, nanoarchitectonics opens ways to form a more unified paradigm by integrating nanotechnology with organic chemistry, supramolecular chemistry, material chemistry, microfabrication technology, and biotechnology [47]. Functional material systems are supposed to be nanoarchitected from these nanoscale units upon the selection and combination of various unit processes including atomic/molecular manipulation, chemical/material conversion, self-assembly/self-organization, field-assisted arrangement, nano/micro fabrication, and biochemical/biological treatment [48]. Since the basic concept of nanoarchitectonics is very general, it can be widely applied to fundamental subjects such as material synthesis [49,50,51] and structure control [52,53,54], as well as to applied fields such as energy [55,56,57], the environment [58,59,60], catalysts [61,62,63], sensors [64,65,66], and devices [67,68,69].

In addition, the concept of nanoarchitectonics has been extended to various bio-based fields. The nanoarchitectonics concept is utilized in a wide range of bio-related areas such as the behavior of biomolecules [70,71], the construction of bioactive material systems [72,73], biomimetic approaches [74,75], artificial enzymes [76], and biomedical applications [77,78,79]. This is because the synthesis of functional materials by nanoarchitectonics is similar in some respects to the construction of functional systems in living organisms. Biological systems consist of the rational organization of constituent molecules. Their structures have highly asymmetric and hierarchical features that allow for chained functional coordination, signal amplification, and vector-like energy and signal flow [80,81]. Such a hierarchical structure is difficult to achieve in artificial systems based on the simple equilibrium process of self-assembly. In contrast, the process of nanoarchitectonics is based on the premise of combining several different processes, which makes it easier to obtain a hierarchical structure. Therefore, nanoarchitectonics is a more suitable methodology for creating highly functional systems based on structural asymmetry and hierarchy like biosystems [82].

Architecture at the nanoscale, which is the basic principle of nanoarchitectonics, has different characteristics from architecture at the microscale and macroscale [83]. Architecture at large scales is less susceptible to disturbances. On the other hand, the interactions between atoms, molecules, and materials at the nanoscale are subject to various perturbations such as thermal fluctuations, stochastic distributions, and quantum effects that are difficult to control but cannot be ignored. At the nano-scale, it is better to think of the various effects as being harmonized under uncertainty, rather than being added together. The creation of materials by nanoarchitectonics should also have these characteristics [84]. In fact, these characteristics are similar to those of functional expression in biological systems. The functional molecules in living organisms are exposed to thermal fluctuations and work to harmonize their functions while undergoing uncontrollable perturbations [85,86]. In this respect, nanoarchitectonics shares some characteristics with biological functional systems.

**Figure 2:**
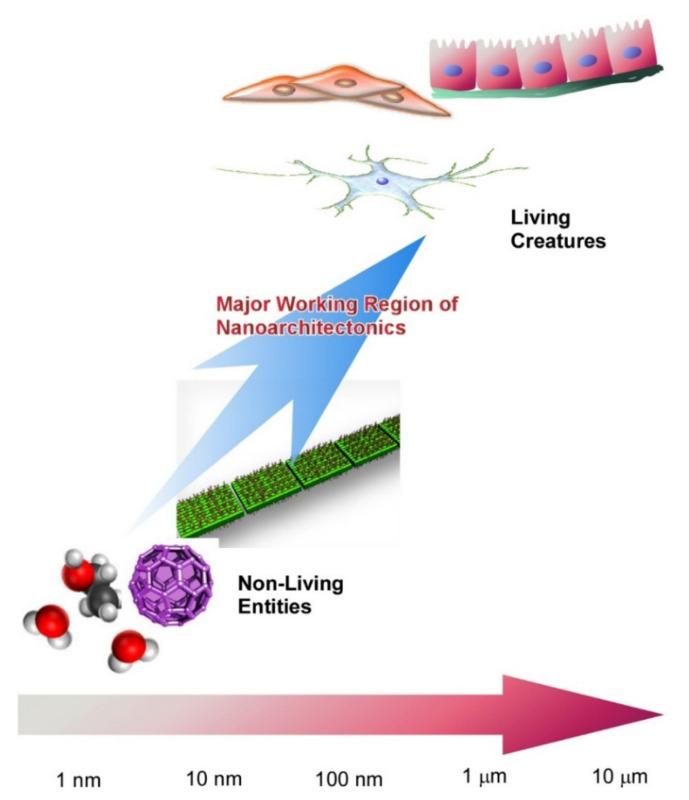
A goal of nanoarchitectonics: creation of highly functional systems similar to those found in biological systems.

The creation of functional materials by nanoarchitectonics is somewhat similar to the creation of functional systems in biological systems. Therefore, it can be said that the goal of nanoarchitectonics is to create highly functional systems similar to those found in biological systems (Figure 2) [87]. The idea of constructing functional systems similar to those of living organisms also has been discussed in biomimetic science [88,89,90] and supramolecular chemistry [91,92,93]. Nanoarchitectonics can integrate such existing fields. Based on these backgrounds, this review article summarizes the synthesis of biomimetic and biological molecules and their functional structure formation from various viewpoints, from the molecular level to the cellular level. Although each of these examples is classified under a pre-existing science category, I believe that they can be reclassified on the basis of nanoarchitectonics. In this review, I have categorized and arranged several recent examples to illustrate such a trend with sections on (i) synthetic nanoarchitectonics for bio-related units, (ii) self-assembly nanoarchitectonics with bio-related units, (iii) nanoarchitectonics with nucleic acids, (iv) nanoarchitectonics with peptides, (v) nanoarchitectonics with proteins, and (vi) bio-related nanoarchitectonics in conjugation with materials. Not all of these examples are well-known, typical or noteworthy, but I have collected those that are in line with the flow of this paper.

## 2. Synthetic Nanoarchitectonics for Bio-Related Units

Bottom-level nanoarchitectonics for biomimetic and biological functional materials can be based on molecular conversions to create complicated functional molecules from rather simple molecular components. This stage of bio-nanoarchitectonics is basically overlapped with activities of organic synthesis for biomimetic and biological molecules. In addition to challenges in the total synthesis of complicated bioactive molecules [94,95], innovative approaches in the organic synthesis of biomolecules have been reported. Yamamoto and co-workers proposed a so-called game changing approach in peptide synthesis with paradigm shifts from reagent-controlled methods to a substrate-controlled strategy [96]. Their method has racemization-free features in Lewis-acid-catalyzed peptide synthesis. While conventional reagent-controlled methods include the formation of highly reactive esters accompanied by occasional racemization upon oxazolone intermediate formation, the substrate-controlled methods proposed by Yamamoto do not cause racemization. The latter is originated from moderate activation by Lewis acids for carbonyl groups with a certain distance from the directing group. Applications of the proposed methods are not limited to peptide synthesis; the strategy can be also utilized in a wider variety of amide molecules including natural products with complicated structures.

Total syntheses of complicated natural products retain their importance in science [97,98]. Although they are categorized as expert organic syntheses, they can be also recognized as molecule-based nanoarchitectonics for bioactive substances. Yoshida, Kigoshi, and co-workers successfully demonstrated the total synthetic approach for hytramycin V, an antibiotic cyclic peptide containing piperazic acid [99]. A cyclization precursor from a hexapeptide was first synthesized, and subsequent macrolactamization of the precursor resulted in the final natural product, hytramycin V.

**Figure 3:**
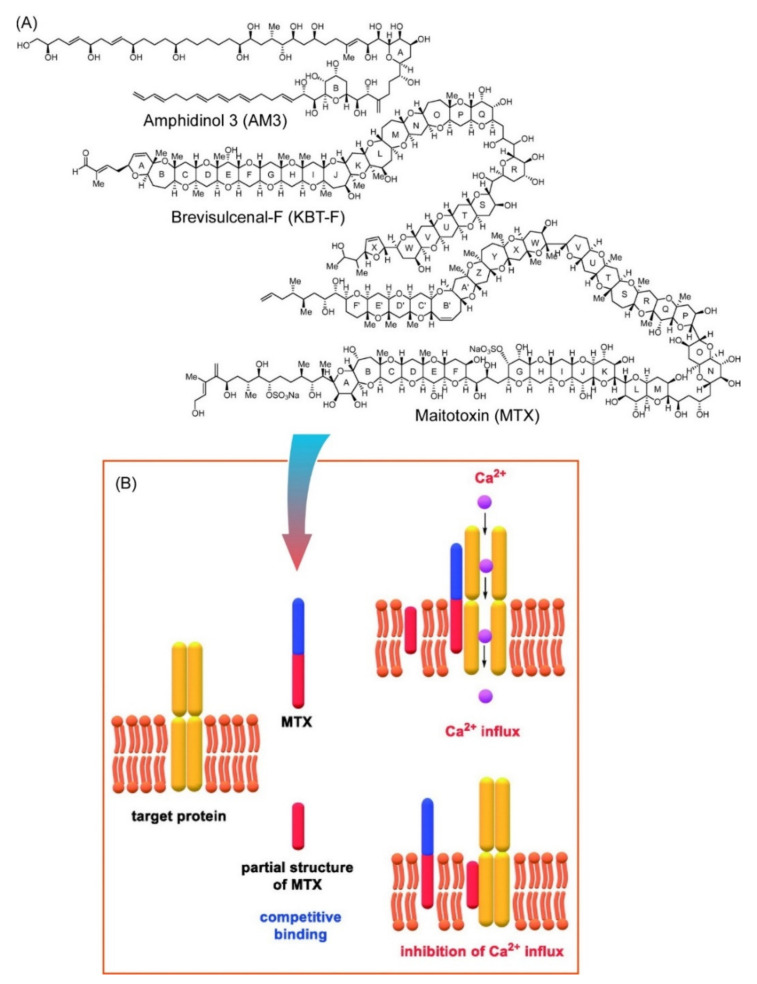
(**A**) Structures of so-called super carbon chain natural products, amphidinol 3 (AM3), brevisulcenal-F (KBT-F), and maitotoxin (MTX). (**B**) Possible inhibition mechanism on Ca^2+^ influx through a cell membrane induced by MTX. Reprinted with permission from Reference [100]. Copyright 2020 Chemical Society of Japan.

Oishi summarized approaches to structure identification, organic synthesis, and biological activities of so-called super carbon chain natural products, amphidinol 3 (AM3), brevisulcenal-F (KBT-F), and maitotoxin (MTX) (Figure 3A), in a recent review article [100]. These super carbon chain compounds are natural products from marine microorganisms with effective antifungal activities and have a long linear backbone with bis-tetrahydopyran, polyol, and polyene moieties. Working principles and a possible inhibition mechanism of Ca^2+^ influx induced by MTX are depicted in Figure 3B. Binding of MTX to hydrophobic parts of target channel proteins buried in a cell membrane induces conformational changes of the target protein with an increase in Ca^2+^ influx through the cell membranes. Shorter analogues of MTX can inhibit the MTX activity through competitive binding. Synthetic approaches on the partial structure mimicking the hydrophobic region of MTX would be important keys to elucidating the mechanisms of MTX activities.

**Figure 4:**
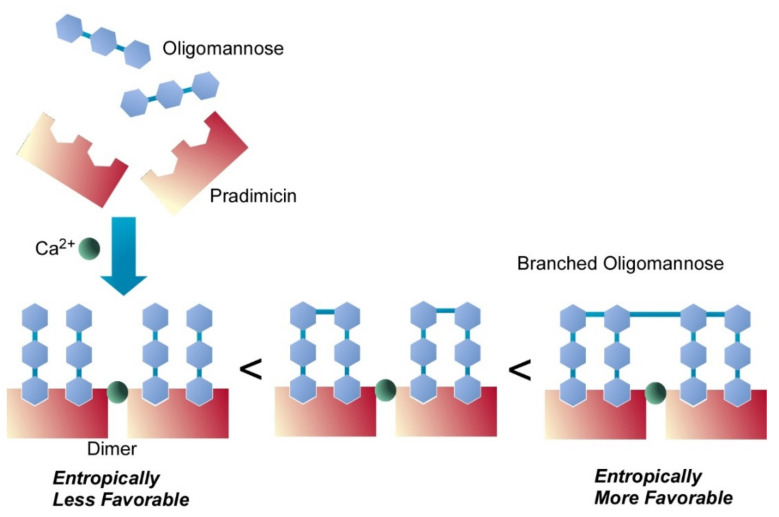
Entropically favorable binding of a branched oligomannose to dimerized pradimicins through binding with Ca^2+^ ions.

Synthetic approaches to nanoarchitect biomimetic molecules are often used in investigations of bioactive phenomena. Nakagawa et al. synthesized various branched oligomannose derivatives that were subjected to evaluation of binding of pradimicins to oligosaccharides [101]. Pradimicins are known as a unique family of natural products with antifungal activity thorough binding with mannans at fungi cell walls. The synthesized oligomannoses mimic the structural motifs of mannans at the cell wall of Candida albicans. Preferential binding of pradimicins to highly branched regions of the fungal mannans was suggested because of the rich presence of terminal mannose residues. As depicted in Figure 4, this preferential binding is also supported by dimerization of pradimicins through binding with Ca^2+^ ions. Entropically favorable binding to highly branched oligomannoses actually resulted in stronger binding of pradimicin to four- and five-branched oligomannoses. Nanoarchitected designs of biomimetic oligosaccharides determine bioactive phenomena.

**Figure 5:**
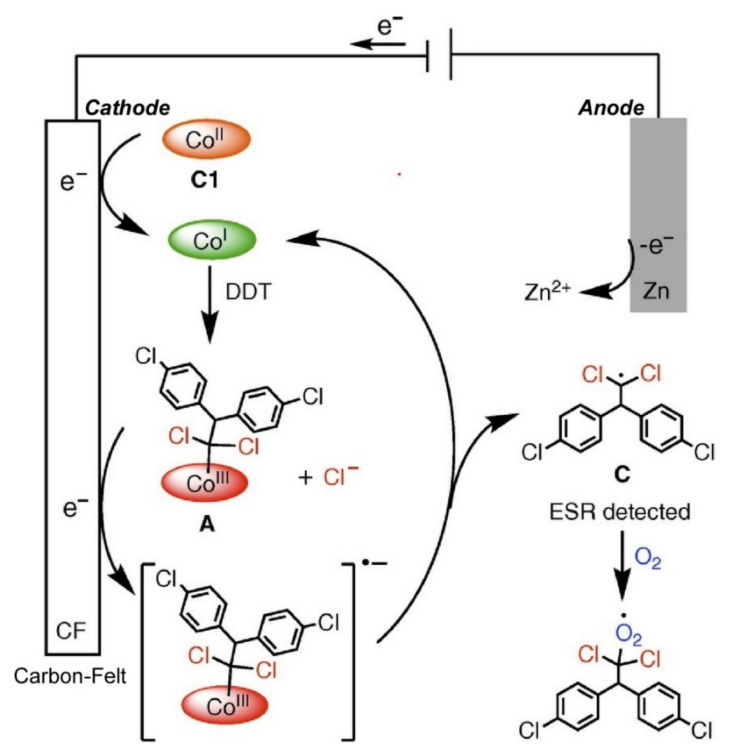
Electrochemical aerobic transformations of 1,1,1-trichloro-2,2-bis(4-chlorophenyl)ethane (DDT) to various oxygen-containing compounds with the aid of catalysis of heptamethyl cobyrinate perchlorate as a vitamin B_12_ derivative. Reprinted with permission from Reference [106]. Copyright 2021 Chemical Society of Japan.

Conjugation of functional molecular units with biological structure motifs also have been widely researched. Uzawa and co-workers successfully demonstrated suppression for quenching of photo-excited ruthenium polypyridyl complexes (4,4′-methylphosphonic acid-2,2′-bipyridine)bis(4,4′-dimethyl-2,2′-bipyridine)ruthenium) by O_2_ through binding with an appropriate RNA aptamer [102]. For various applications including light energy conversion to chemicals and/or electric energy, ruthenium polypyridyl complexes have been widely investigated [103,104]. In these cases, deactivation of triplet metal-to-ligand charge transfer excited states by O_2_ quenching has to be minimized. Binding of the designed RNA aptamer can ideally suppress the O_2_ quenching of the ruthenium complex. Charge transfer from sacrificial donor components to the ruthenium complex bound to the RNA aptamer was promoted. Nanoarchitectonics strategy between the ruthenium complex and RNA aptamer is a promising way to retain efficient photoelectrochemical functions. Tamiaki and coworkers successfully conjugated chlorophyll-a derivatives with electron-donating *p*-dimethylamino groups [105]. Higher quantum yields and longer lifetimes of fluorescence emission were observed because of suppression of fluorescence quenching upon intramolecular electron-transfer. Simple substitution with aminopyridines is an effective way to nanoarchitect highly fluorescent chlorophyll-a derivatives. Nanoarchitectonics-based immobilization of co-enzymes onto electrodes works with electrochemical molecular transformation, as recently demonstrated by Hisaeda, Shimakoshi, and co-workers [106]. As illustrated in Figure 5, electrochemical aerobic transformations of 1,1,1-trichloro-2,2-bis(4-chlorophenyl)ethane (DDT) to various oxygen-containing compounds were achieved with the aid of catalysis of heptamethyl cobyrinate perchlorate as a vitamin B_12_ derivative. The demonstrated electrochemical conversion systems can lead to the utilization of environmental pollutants for the production of useful organic compounds.

**Figure 6:**
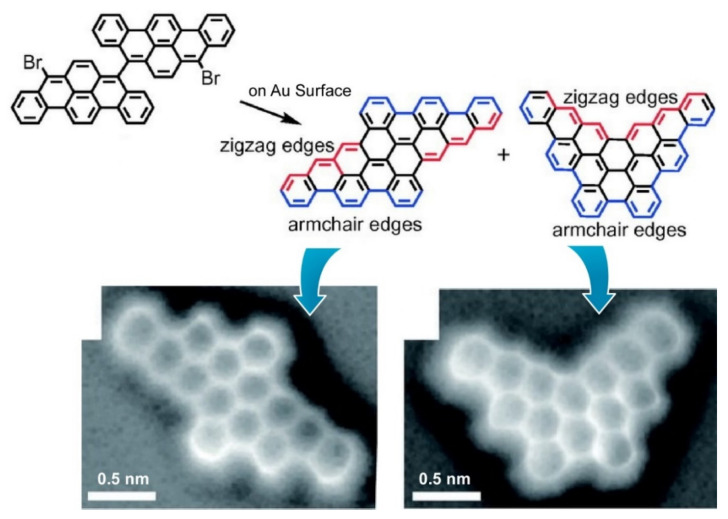
On-surface synthesis and high-resolution observation for parallelogram-shaped dibenzohexacenohexacene (**left**) and butterfly-shaped dibenzopentaphenoheptaphene (**right**) possessing armchair and zigzag edges. Reprinted with permission from Reference [113]. Copyright 2021 Chemical Society of Japan.

As illustrated above, organic synthesis has crucial roles in the nanoarchitectonics of biomimetic and biological molecules. Much deeper couplings between organic synthesis and nanotechnology have been recently reported. So-called local probe chemistry enables us to perform point-by-point chemical reactions using a scanning microscopy probe tip [107,108]. For example, point-substitution of Br atoms on graphene nanotape by a fullerene molecule was demonstrated with the probe tip of a scanning probe microscope by Kawai and coworkers [109]. Removing Br atoms from specific positions on the graphene nanotape and attaching fullerene molecules to the reacted point can be achieved with the actions of the probe tip. Unlike usual organic synthesis with certain selectivity and specificity, reaction points are designable with nanotechnological tools. This is a nice demonstration of molecular-level nanoarchitectonics based on a fusion of nanotechnology and organic synthesis. Combinations of nanotechnological atom/molecular-level observations and organic syntheses also have been researched as approaches to on-surface synthesis [110,111]. Müllen, Narita, and co-workers demonstrated several examples for the on-surface synthesis of two-dimensional graphene pieces with defined shapes and sizes [112]. On-surface synthesis and high-resolution observation for butterfly-shaped dibenzopentaphenoheptaphene and parallelogram-shaped dibenzohexacenohexacene possessing armchair and zigzag edges were actually reported (Figure 6) [113]. Such strategies based on the combination of nanotechnology and organic syntheses can be generalized to a wide range of molecules, including biomimetic and biological molecules. In the near future, bioactive molecules will be nanoarchitected according to exact designs using nanotechnology-driven organic syntheses.

## 3. Self-Assembly Nanoarchitectonics with Bio-Related Units, General

Well-synthesized molecular units can be nanoarchitected into one-step higher organization upon molecular self-assembly. Most of the important functional systems in biology are formed by the self-assembly/self-organization of component biomolecules. As seen in cell membranes with lipids, proteins, and oligosaccharides, protein architectures with peptide segments, and formation of gene-active structures with DNA and RNA, bio-related molecules rationally assemble into functional structures through specific interactions such as hydrophobic effects, hydrogen bonding, and electrostatic interaction [114,115,116]. Based on this working principle of biomolecular assembly, self-assembly nanoarchitectonics to fabricate functional materials and structures are being widely researched using biomimetic molecules and bioconjugate components [117,118,119]. For example, functions based on helical organizations from biomolecules and synthetic polymers have been investigated mainly since the early 1950s. As stated in a recent review article by Percec and Xiao [120], a wide variety of biological molecules including proteins, nucleic acids, carbohydrates, and their synthetic mimics are subjected to the formation of helical structures, as seen in the beautiful helical co-assembly of proteins and nucleic acids in the tobacco mosaic virus.

**Figure 7:**
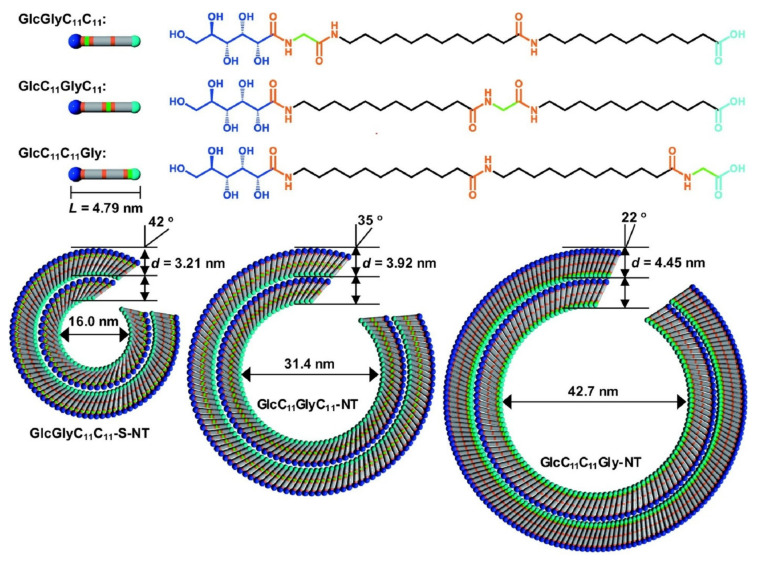
Formation of nanotubes with different inner diameters upon self-assembly of three asymmetrical bolaamphiphile-type glycolipids having common moieties of glycine, 12-aminododecanoic acids, and D-gluconolactone and different connection sequences. Reprinted with permission from Reference [126]. Copyright 2021 Chemical Society of Japan.

Amphiphile molecules including lipids and surfactants with an appropriate balance between hydrophilic groups and hydrophobic moieties universally form bilayer structures such as cell membranes (so-called liposomes and vesicles) [121,122]. Further structural manipulation of component molecules leads to the formation of non-vesicular structures. For example, formations of tubular nanoarchitectures are achieved through the self-assembly of biomimetic lipid-like component molecules [123,124,125]. Recently, Kameta and co-workers reported the preparation of supramolecular lipid nanotubes with well-controlled diameters (Figure 7) [126]. In their approach, three asymmetrical bolaamphiphile-type glycolipids with common moieties of glycine, 12-aminododecanoic acids, and D-gluconolactone and different connection sequences were used as nanotube components. These glycolipids self-assemble in water to yield nanotubes with different inner diameters, which are deeply related with the strength of intermolecular hydrogen bonding and intensity of supramolecular chirality. Small inner diameters of the supramolecular tubes are generally achieved when intermolecular hydrogen bonds are weak, and the tilt angles of the component molecules with unit layers are large upon intense chirality. Controllable nanoarchitectonics on the inner diameters of supramolecular nanotubes would be useful for the regulation of controlled release of entrapped functional molecules such as drugs.

Various biomimetic components can be subjected to self-assembly nanoarchitectonics. Hata and Serizawa reported the fabrication of robust gels upon self-assembly of cello-oligosaccharide networks [127]. Based on extremely low responsiveness of the formed gels to external stimuli, the prepared gels exhibit solvent exchange features even from water to nonpolar solvents. This solvent-tolerant nature of the gels could lead to various unconventional applications. Versatile nanomaterial confinement within the gels under incompatible environments, such as confinement of hydrophobic nanocarbons in water, becomes possible. The introduction of growth factors and cell-adhesion peptides of these oligosaccharide gels is a useful way to fabricate cell culture matrices. Partial degradation of the gels makes them water-soluble for further applications.

**Figure 8:**
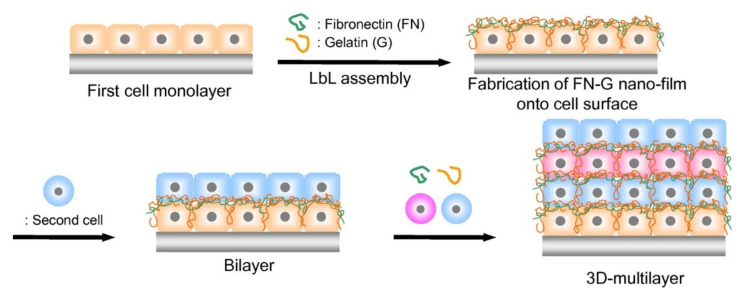
Three-dimensional nanoarchitectonics of living cells through their LbL assembly with the aid of fibronectin and gelatin. Reprinted with permission from Reference [132]. Copyright 2021 Chemical Society of Japan.

Nanoarchitectonics with biomimetic and biological molecules are not limited to spontaneous self-assembly processes. Process-included self-assembly methods such as layer-by-layer (LbL) assembly are also powerful methodologies for bio-active assemblies [128,129,130]. LbL assembly using bioactive components and interactive components (polymers in most cases) can be used as a promising method for multilayer-type nanoarchitectonics of biomimetic and biological components. Based on this strategy, multilayer nanoarchitectures can be fabricated on living cells [131], and even living cells can be used as components of multilayer nanoarchitectures. As explained in a recent review article by Akashi and Akagi [132], LbL assemblies of living cells are capable of forming functional three-dimensional tissues, which can be used as three-dimensional skin models and three-dimensional heart models. As depicted in Figure 8, living cells are three-dimensionally nanoarchitected through their LbL assembly with the aid of fibronectin and gelatin. Although fundamental mechanisms of these living cell strategies are based on basic LbL nanoarchitectures with basic physicochemical and biochemical interactions, the output structures can be applicable to advanced fields of regenerative medicine.

## 4. Nanoarchitectonics with Nucleic Acids

**Figure 9:**
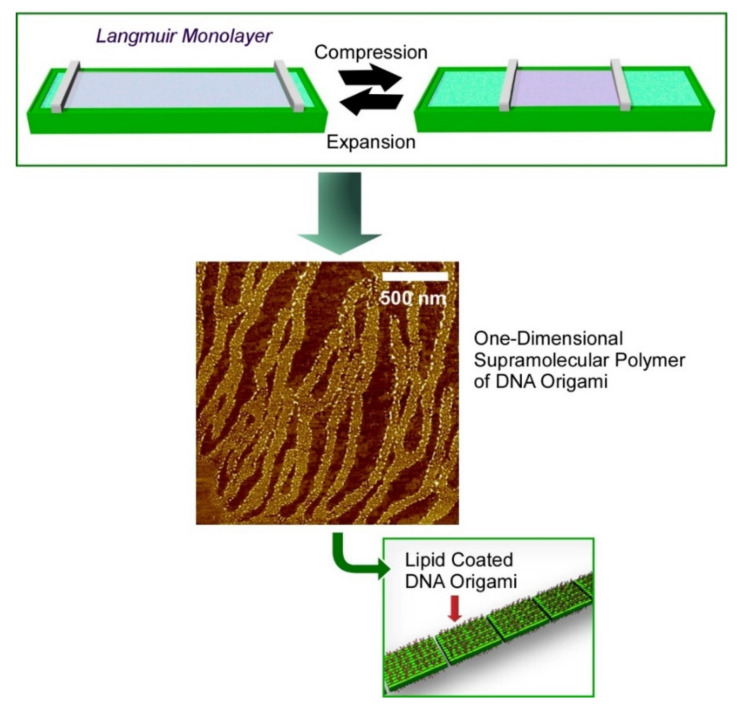
Formation of one-dimensional supramolecular polymers through dynamic mechanical motions of monolayer compression and expansion of a Langmuir film of lipid-coated DNA origami pieces at the air–water interface.

In the following sections, self-assembly nanoarchitectonics of biomimetic and biological molecules are discussed with classifications of typical bioactive motifs, i.e., nucleic acids, peptides, and proteins. Nanoarchitectonics examples with nucleic acids (mainly DNA and RNA) as the first category are discussed below. As compared with the other bioactive motifs such as peptides and oligosaccharides, nucleic acids follow well-defined modes of self-assembly on the basis of strict base pairing (molecular recognition) through complementary hydrogen bonding [133,134]. Self-assembling motifs are not limited to standard double helix formation from two strands. Non-double strand helix and branched assemblies from more than two strands can be formed where strict base pairing is, however, retained as the principal mechanism. These strong features of nucleic acids are used for the formation of well-programmed nanoarchitectures, as seen in DNA origami approaches. As illustrated in Figure 9, hierarchical assemblies of DNA origami pieces are nanoarchitected through self-assembly and interfacial processes [135]. DNA origami pieces programmed for two-dimensional rectangle shapes were first modified with cationic lipid molecules to gain sufficient hydrophobicity for the capability of forming a Langmuir film at the air–water interface. Lipid-coated DNA origami pieces floatin on water were self-assembled into one-dimensional supramolecular polymers through the dynamic mechanical motions of monolayer compression and expansion. This example strikingly explains the strong potential to form hierarchical nanoarchitectures using programmed DNA strands as components.

**Figure 10:**
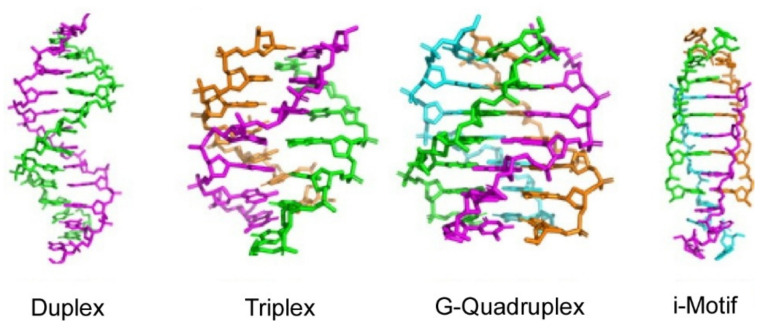
Non-double helix (non-canonical) DNA nanoarchitectures such as triplexes, G-quadruplexes, and i-motifs. Reprinted with permission from Reference [136]. Copyright 2021 Chemical Society of Japan.

Structural motifs adopted by DNA association are not limited to typical double helix nanoarchitectures. Various non-double helical motifs also have been considered as functional structures. Sugimoto et al. discussed chemical and biological perspectives on non-double helix (non-canonical) nanoarchitectures such as triplexes, G-quadruplexes, and i-motifs (Figure 10) [136]. Stability of the non-canonical motifs is enhanced under molecular crowding conditions. Molecular crowding conditions are mainly present in intracellular environments with dense packing of various molecules. Roles of the noncanonical motifs in gene expression were also discovered together with recognition of the importance of molecular crowding environments in cells for functionalized biomolecules.

**Figure 11:**
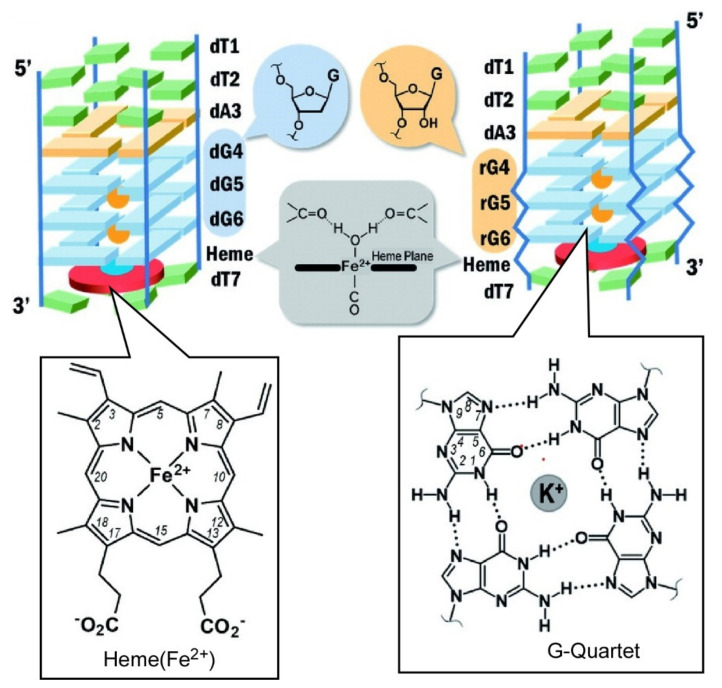
All-parallel tetrameric G-quadruplex consisting of a DNA/RNA chimera sequence for incorporation of heme units. Reprinted with permission from Reference [140]. Copyright 2020 Chemical Society of Japan.

G-quadruplexes are used for nanoarchitectonics of functional structures. A type of higher-order structure of DNA and RNA, the G-quadruplex is a quadruplex structure formed by guanine-rich nucleic acid sequences [137,138,139]. The G-quadruplex structure is found in telomeres and is thought to be involved in the regulation of gene expression and telomerase. Yamamoto and co-workers nanoarchitected an all-parallel tetrameric G-quadruplex consisting of a DNA/RNA chimera sequence for incorporation of heme units (Figure 11) [140]. The G-quadruplex with chimera sequence is especially stabilized through interstrand hydrogen bonds and provides heme accommodation space at the 3-terminal of the G-quartet. In G-quadruplex nanoarchitectures, peroxidase activity of the bound heme is not affected by the 2.-OH group of the ribose in RNA strands. Substrate binding is regulated within confined complex nanospace. A CO molecule can bind to the heme Fe atom from the opposite side of the G-quadruplex, and an H_2_O molecule is coordinated to the Fe atom from the G-quadruplex side as another axial ligand. The same research group also investigated photogeneration of reactive oxygen species from phthalocyanine derivatives immobilized in the all-parallel tetrameric G-quadruplex [141]. Photogeneration of reactive oxygen species from Zn-complexed phthalocyanine derivative is enhanced in the presence of the DNA motifs. The observed enhancement is probably originated from dissociation of the non-fluorescent aggregate upon interaction with the DNA. The latter feature indicates interaction of photosensitizer with DNA and/or RNA would be beneficial for photodynamic therapy.

The strong ability to create precise nanoarchitectures of programmed DNA/RNA nanoarchitectonics is an advantageous feature for the formation of interlocked supramolecular structures, as summarized in a recent review article by Liang et al. [142]. Nanoarchitectonics of various supramolecular structures were demonstrated to create DNA/RNA-based catenane and rotaxane structures that could be further elaborated for the formation of nanoshuttles, nanowalkers, molecular transporters, nanopumps, nanorobots, logic gates, and amplifiers. These supramolecular DNA/RNA structures would be connected with various functions such as sensing and catalysis. Liang, Komiyama, and co-workers also discussed the importance of ring-architecture DNA and RNA including naturally occurring rings such as circulating cell-free DNAs, cyclic RNAs, and extrachromosomal circular DNA and artificial DNA/RNA rings synthesized from oligonucleotide fragments [143]. The latter artificial rings can be nanoarchitected using DNA ligases with short oligonucleotides prepared by automated DNA synthesizers.

**Figure 12:**
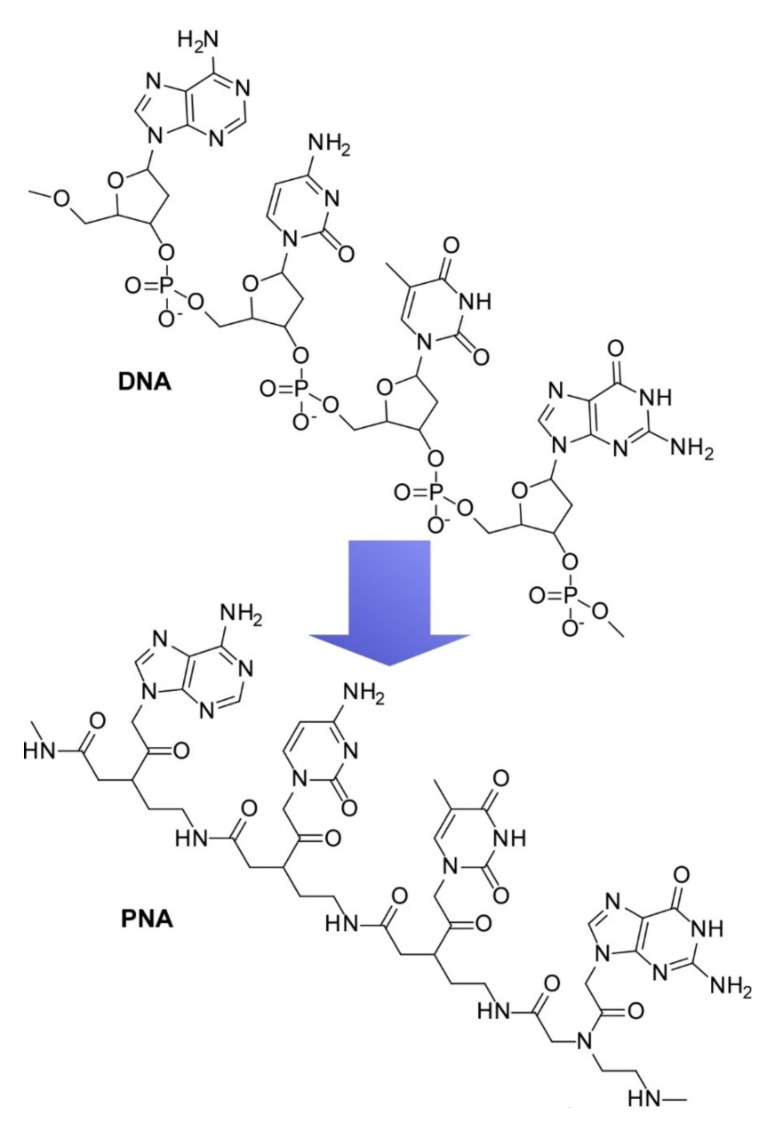
A peptide nucleic acid (PNA) as an artificial mimic of DNA.

As artificial mimics of DNA, peptide nucleic acids (PNAs) also have been investigated to produce functional materials [144,145]. PNA as a DNA analogue possesses poly[N-(2-aminoethyl)glycine] instead of the phosphate–sugar backbone of DNA strands (Figure 12). PNA strands are capable of sequence-specific recognition of DNA and RNA in the forms of single strands, double strands, i-motifs, G-quadruplexes, and so on. Complex formation with DNA and RNA induces the structural constraint of PNA strands, resulting in the designed transformation of target sites. This dynamic structural regulation can be used for control of gene expression and gene editing. In a recent review article [146], Sato summarized the development of PNA-based synthetic fluorescent probes having a fluorogenic cyanine chromophore, which are designed for targeting the overhang motifs of double-strand RNAs in investigations of the processes of intracellular delivery of small interfering RNAs. Triplex formation between PNA probes and RNA enables the sequence-selective detection of double-strand RNAs. Fluorescent probes with double-strand RNA targeting capability are effectively used for evaluation of complicated RNA functions within cellular environments. Kim and co-workers comprehensively summarized progress on fluorescent nucleic acid systems for biosensors [147]. The development of quencher-free molecular aptamer beacons, three-way junction probes, and probes for G-quadruplexes and i-motifs would afford considerable benefits to evaluations of various biological events.

**Figure 13:**
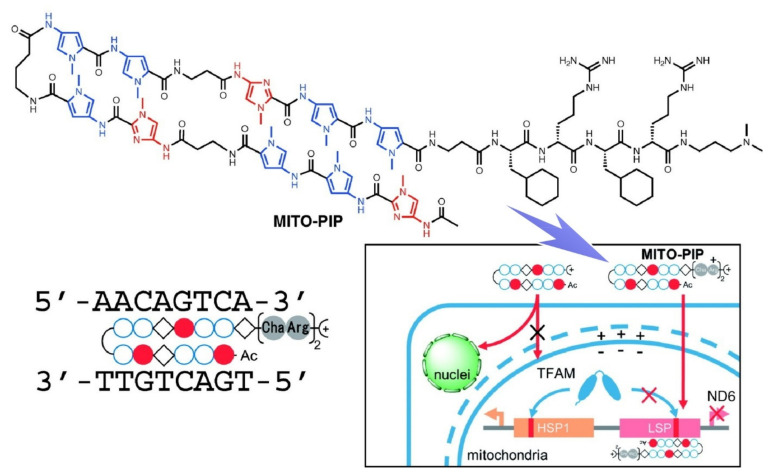
Peptide sequence with cyclohexylalanine and arginine attached to the polyamide for regulation of the expression of mitochondrial DNA within mitochondria. Reprinted with permission from Reference [148]. Copyright 2020 Chemical Society of Japan.

Polyamides with the ability to form hydrogen bonds with DNA structures are used for DNA analyses, as described by Bando and Sugiyama [148]. The polyamides were designed to have the ability to bind to DNA minor grooves at their N-methylpyrrole and N-methylimidazole groups. With this molecular architecture, regulation of specific gene expression and visualization of specific DNA sequences in cells become possible on the basis of specific sequence recognition. Among the demonstrated targets are eukaryotic organelles having mitochondrial DNAs that are known to regulate ATP synthesis for energy production. A peptide sequence with cyclohexylalanine and arginine was attached to the polyamide for promotion of penetration into mitochondria (Figure 13). Regulation of the expression of mitochondrial DNA within mitochondria was examined. Treatment with the nanoarchitected polyamide with targeting capability to the light-strand promoter (LSP) region in the mitochondrial transcription factor A (TFAM)-binding sequence in HeLa cells resulted in suppression of the expression of the downstream gene through selective binding of the polyamide to TFAM.

## 5. Nanoarchitectonics with Peptides

**Figure 14:**
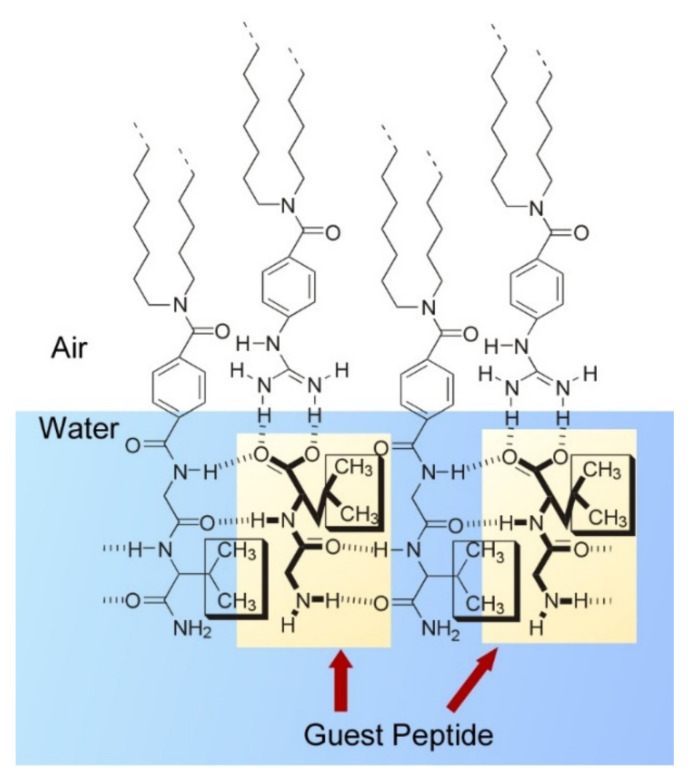
Monolayers of simple dipeptide molecules with hydrophobic tails at the air–water interface forming molecular-size spaces for specific recognition of aqueous guest dipeptides.

**Figure 15:**
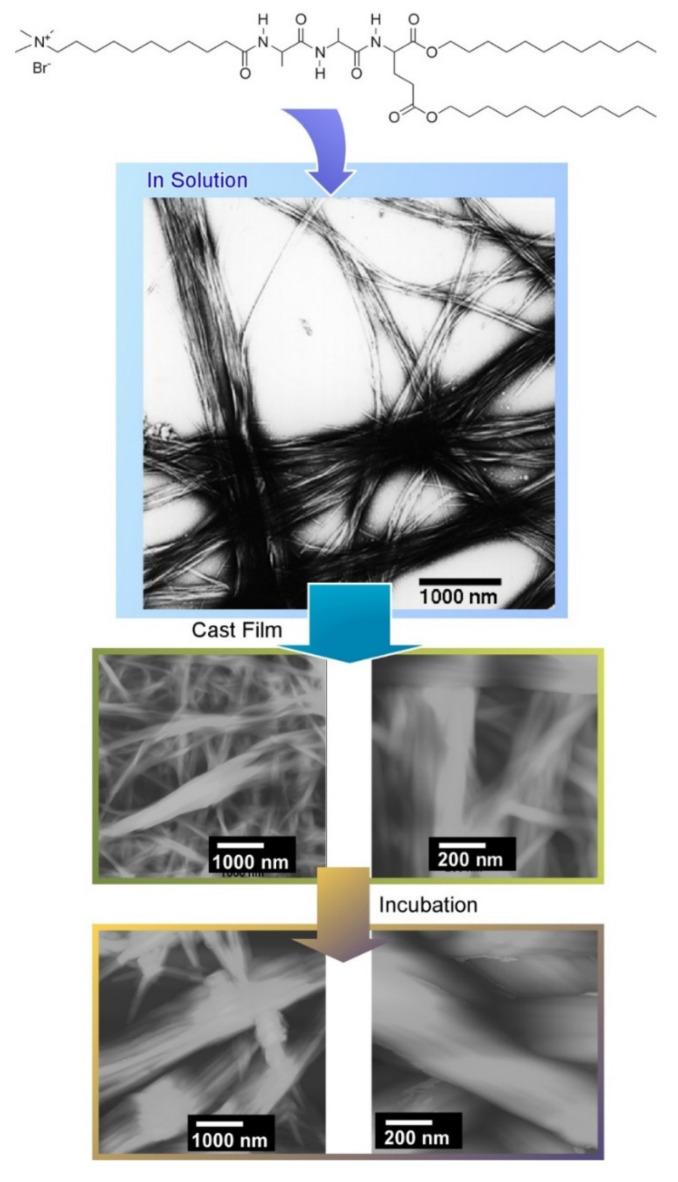
Hierarchic fibrous structures formed in solutions and cast films with molecules with tripeptide hydrogen bonding moiety within amphiphilic lipid motif.

Although peptide sequences do not generally possess recognition capability for highly specific pairing like DNA and RNA, their strong capability of hydrogen bond-based assembly formation is a crucial factor in many important biological events such as protein folding and substrate discrimination. With this strong capability of structure formation, peptide units have been used for active components in self-assembly nanoarchitectonics [149,150]. Even short peptides such as dipeptides and tripeptides can form regular nanoarchitectures with specific molecular recognition and polymorphic structure formation through coupling with hydrophobic moieties and/or hydrophilic groups. For example, simple dipeptide molecules with hydrophobic tails can form regular monolayer structures at the air–water interface [151,152,153]. Molecular-size spaces nanoarchitected at the surface can specifically recognize aqueous guest dipeptides (Figure 14). Various factors including parallel/antiparallel beta-sheet-like hydrogen bonding, side-chain steric hindrances, interaction at terminal groups and positions of hydrophobic residues determine efficiencies of guest peptide binding. This is a nice mimic of naturally occurring receptors and enzyme pockets. By combining the hydrogen bonding capabilities of short peptides and self-assembling behaviors of amphiphilic molecules, a variety of modulations of the assembled nanoarchitectures can be realized [154]. Hierarchic fibrous structures are formed in solutions and cast films with molecules with tripeptide hydrogen bonding moiety with amphiphilic lipid motif (Figure 15). Alteration of peptide sequences and casting solvents would create various possibilities in the resulting nanoarchitectures in their cast films, as seen in the formation of thick fibers, thin needles, and two-dimensional patterns. Not limited to these examples, short peptides such as dipeptides and tripeptides are widely used for the formation of regular assemblies for various functions including biomedical applications [155,156].

**Figure 16:**
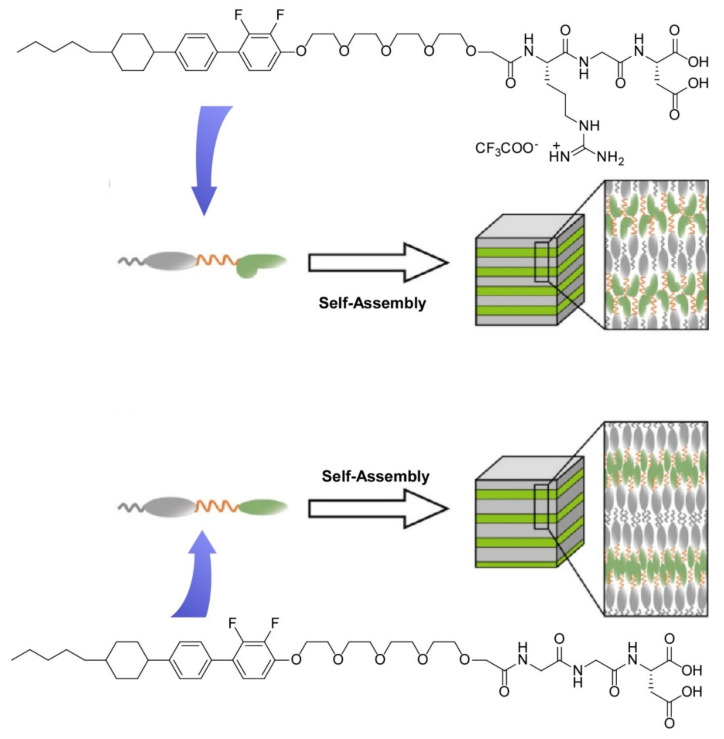
Steric effects of peptide side chains in molecular assemblies of liquid crystalline phase through synthesizing mesogens with arginine-glycine-aspartic acid part (top) and glycine-glycine-aspartic acid part (bottom). Reprinted with permission from Reference [157]. Copyright 2021 Chemical Society of Japan.

Kato and co-workers investigated steric effects of peptide side chains in molecular assemblies in liquid crystalline phase by synthesizing mesogens with three kinds of tripeptides: arginine-glycine-aspartic acid, glycine-glycine-aspartic acid, and triglycine [157]. These peptide moieties are connected to a rigid-rod core via a flexible spacer of tetraoxyethylene. Intermolecular hydrogen bonds are formed at the tripeptide parts in the liquid crystalline phase that can be aligned by mechanical shearing, because the strength of the intermolecular hydrogen bonding is significantly affected by steric hindrance at the peptide side chains. As depicted in Figure 16, a nano-segregated nanoarchitecture between the arginine-glycine-aspartic acid parts and the rigid cores is formed with certain interdigitation at the rigid core moieties. In case of less bulky glycine-glycine-aspartic acid peptide, a less interdigitated layer of the rigid core layers can be formed. Faint differences in peptide residues would affect molecular packing in assembled structures and thus regulate their liquid crystalline properties.

**Figure 17:**
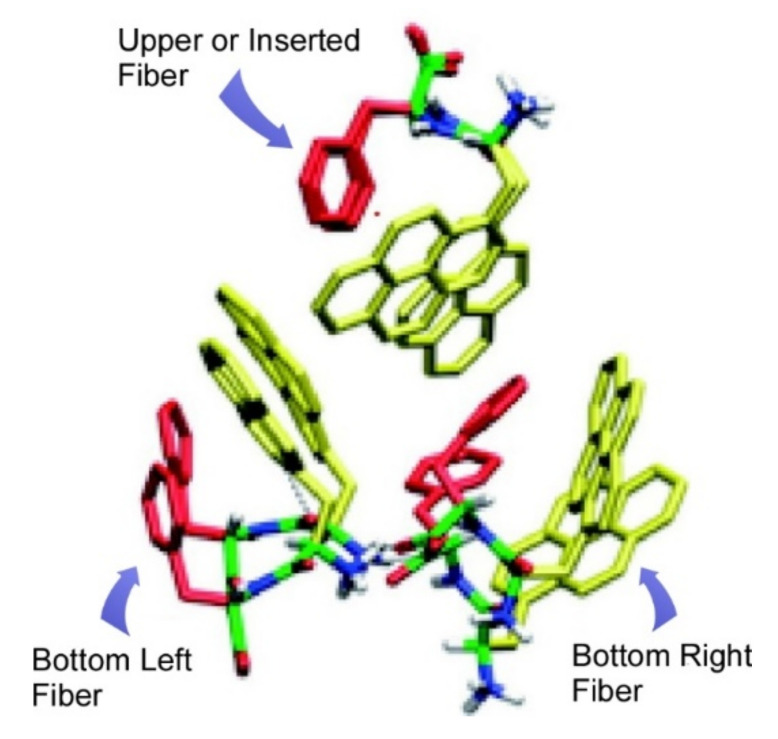
Pyrene–pyrene interactions between two D-pyrenylalanine-L-phenylalanine dipeptide fibers as suggested by molecular simulation. Reprinted with permission from Reference [158]. Copyright 2020 Chemical Society of Japan.

Stereochemical effects on molecular assemblies of dipeptides of pyrenylalanine-phenylalanine dipeptide were investigated by Kawamura and co-workers [158]. While L-pyrenylalanine-L-phenylalanine is self-assembled into solid fiber structures, hydrogels are formed upon self-assembly of D-pyrenylalanine-L-phenylalanine. With fluorescence spectroscopy, monomer emission was observed for L-pyrenylalanine-L-phenylalanine assembly, but D-pyrenylalanine-L-phenylalanine assembly revealed red-shifted excimer emission. Molecular simulation indicated pyrene–pyrene interactions between two D-pyrenylalanine-L-phenylalanine dipeptide fibers (Figure 17). Only one point difference of stereochemistry results in drastic differences in photonic properties. Further understanding of stereochemical effects in peptide nanoarchitectonics will open up various possibilities for the use of peptide assemblies in various applications.

**Figure 18:**
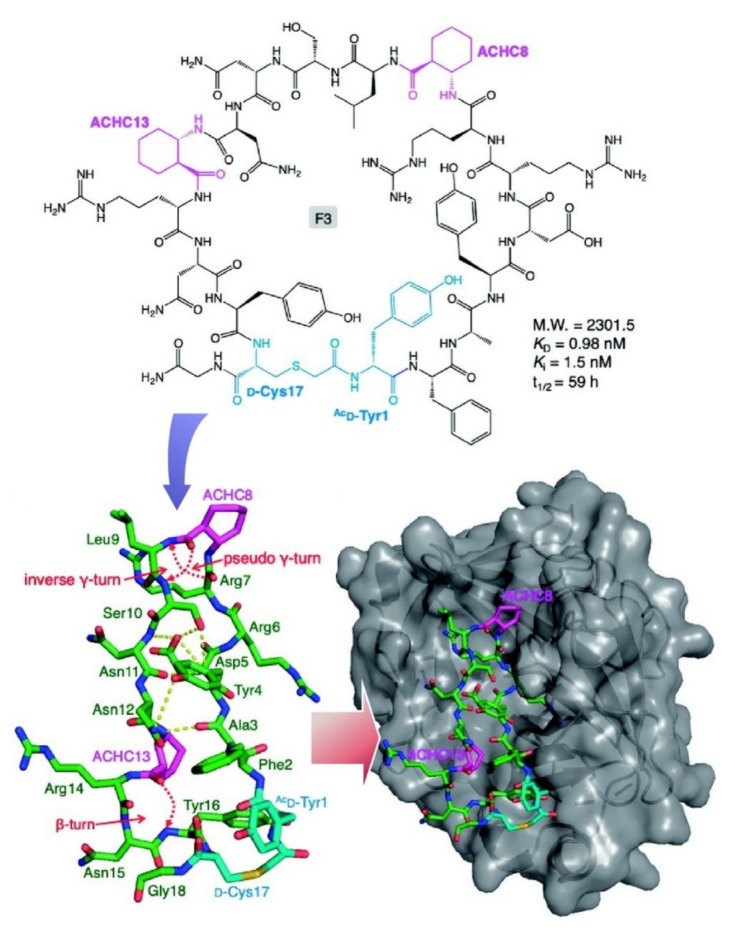
Selected peptide binders to the human contact activation protease factor XIIa (FXIIa) with binding based on logical folding of the binder proteins through formation of anti-parallel β-sheet structure supported by two γ-turns, a pseudo γ-turn and an inverse γ-turn. Reprinted with permission from Reference [160]. Copyright 2021 Chemical Society of Japan.

Peptide assemblies in biological systems sometimes cause fatal diseases, as seen in the misfolding and aggregation of amyloid β peptide and Tau protein. For the diagnosis of Alzheimer’s disease, interactive molecules with toxic polymorphic peptide species have been investigated for detection and imaging of Alzheimer’s disease biomarkers, as summarized in the recent review article by Govindaraju and co-workers [159]. Multimodal detection and imaging are keys for the future of Alzheimer’s disease diagnostics. For parallel and ratiometric analyses, the development of nice sets of target-specific molecular probes is indispensable. Katoh and Suga used genetic code manipulation nanoarchitectonics to produce peptide libraries through ribosomal synthesis with a wide range of nonproteinogenic amino acids [160]. The prepared libraries can be used for the discovery of bioactive peptides with the aid of screening methods such as mRNA display techniques. Figure 18 illustrates selected peptide binders to the human contact activation protease factor XIIa (FXIIa). Co-crystallization structural analyses revealed logical folding of the binder proteins through formation of an anti-parallel β-sheet structure supported by two γ-turns, a pseudo γ-turn and an inverse γ-turn. Intramolecular van der Waals with side chains also have important roles in peptide folding and binding. Library-based peptide nanoarchitectonics would be an effective way to find the best solutions from numerous possibilities.

**Figure 19:**
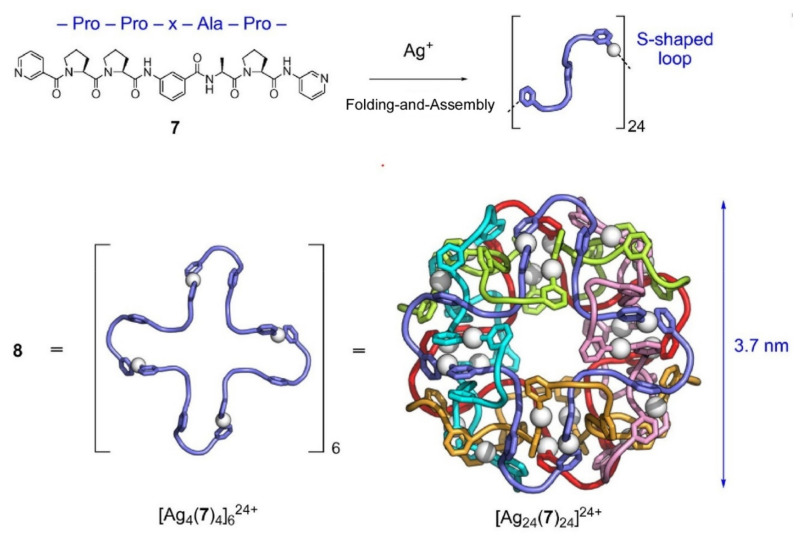
Formation of huge capsule of 3.7 nm in size with a cubic shape with 24-crossings, three-crossing structure, doubly twist structure, and huge hollow structure of ca 3.2 nm^3^ inner volume from Pro-Pro-x-Ala-Pro peptide sequence and Ag^+^ ions. Reprinted with permission from Reference [161]. Copyright 2021 Chemical Society of Japan.

Like DNA and RNA, peptide strands can be used for the formation of entangled supramolecular nanoarchitectures such as polyhedral links, torus knots, and a poly[n]catenane, as demonstrated by Sawada and Fujita [161]. Both the metal-based self-assembly and peptide self-folding work on formation of topological nanoarchitectures upon threading of multiple metal-peptide rings. As depicted in Figure 19, Pro-Pro-x-Ala-Pro peptide sequence and Ag^+^ ions provide a huge capsule of 3.7 nm in size with a cubic shape with 24-crossings, three-crossing structure, doubly twist structure, and huge hollow structure of ca 3.2 nm^3^ inner volume. Modification of the inner surface of the capsule structures is also possible simply by replacing peptide components with a Pro-Pro-x-Cit-Pro sequence (Cit: L-citrulline). The newly nanoarchitected capsule can accommodate 1,3,5-benzenetriacetic acid with the aid of hydrogen bond formation at the capsular interior surface.

**Figure 20:**
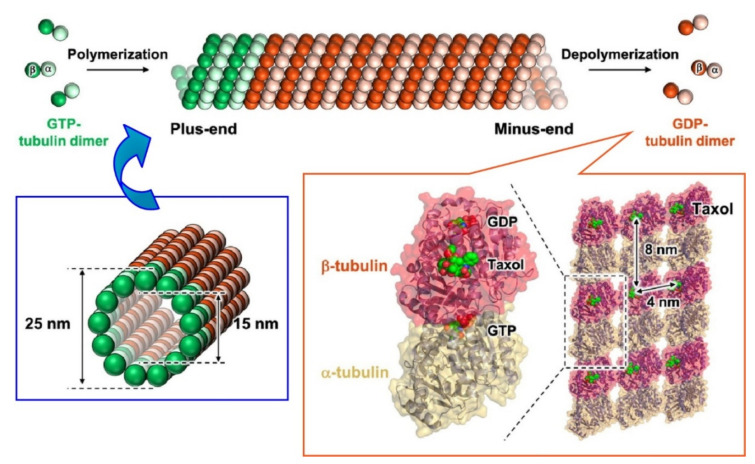
Microtubules are known as hollow cytoskeletons formed through the formation of linear protofilaments upon head-to-tail self-assembly of tubulin dimers, subsequent sheet structure formation, and finally closing of the formed sheets. Reprinted with permission from Reference [162]. Copyright 2021 Chemical Society of Japan.

Inaba and Matsuura demonstrated property modulation of microtubules upon encapsulation of nanomaterials with the aid of Tau-derived peptides [162]. Microtubules are known as hollow cytoskeletons (Figure 20) that are formed through the formation of linear protofilaments upon the head-to-tail self-assembly of tubulin dimers, subsequent sheet structure formation, and finally closing of the formed sheets. Nanoarchitected microtubules typically have an inner diameter of 15 nm and play important roles in various biological events in cells such as transport, structural support, and cell division. Because functionalization of the interior environment of microtubules is highly possible, modifications of the inner surface by adsorption of Tau-derived peptides were performed for molecular encapsulation. The properties and functions of microtubules are altered by encapsulation of functional units. For example, photoactive functions can be nanoarchitected through the functionalization of azo-benzene units. Various photosensitive properties have been utilized in applications such as photo-switchable microtubule-binding drugs [163], azobenzene-based photo-switchable inhibitors of a mitotic kinesin [164], and optogenetic activities [165].

## 6. Nanoarchitectonics with Proteins

**Figure 21:**
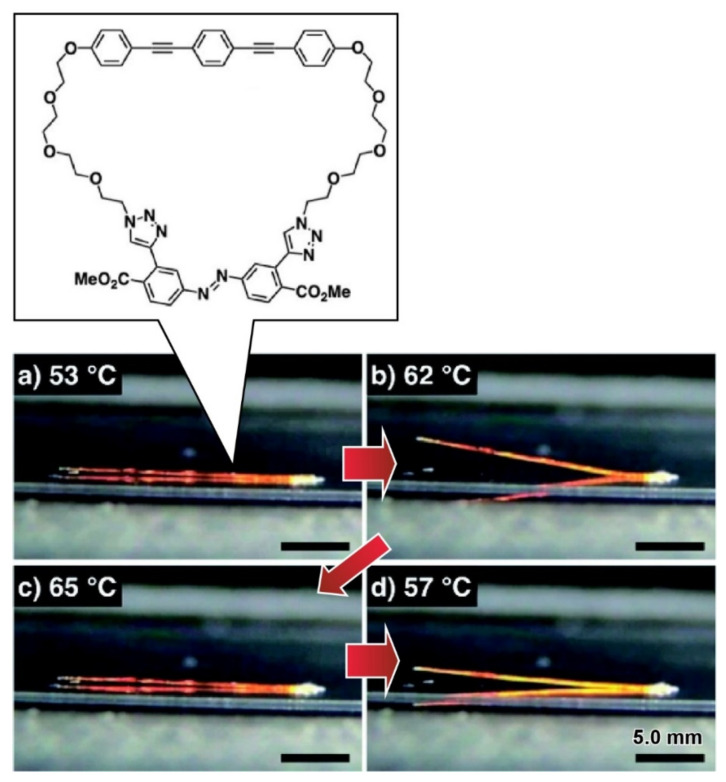
Single crystals with needle shapes formed with the macrocyclic compound with two tetra(ethylene glycol)s, bis(phenylethynyl)benzene moiety, and trans-azobenzene unit with reversible bending motions upon polymorphic transition between single crystal states upon heating. Reprinted with permission from Reference [166]. Copyright 2020 Chemical Society of Japan.

The development of protein mimicry molecules and the functional modulation and assembly of proteins themselves also have been researched, which may be regarded as a nanoarchitectonics approach with proteins. As a protein mimicry approach with totally synthetic molecules, Muraoka summarized the use of amphiphilic long-chain molecules and macrocyclic compounds with poly(ethylene glycol) moieties in his recent review article [166]. Some of the synthesized protein mimicry molecules exhibited functions similar to those of ion channels in lipid bilayer membranes. Ion transport can be regulated in response to external stimuli. Living-creature-like self-motions were also achieved with the related macrocyclic compound with two tetra(ethylene glycol)s, bis(phenylethynyl)benzene moiety, and trans-azobenzene unit. Single crystals with needle shapes formed with this macrocyclic compound exhibited reversible bending motions upon polymorphic transition between single crystal states (Figure 21). Upon heating, the prepared crystals started bending at 52.3 °C and returned to straight shapes at 60.5 °C. These processes are reversible. Totally non-biological molecules can mimic muscle-like bionic motions.

**Figure 22:**
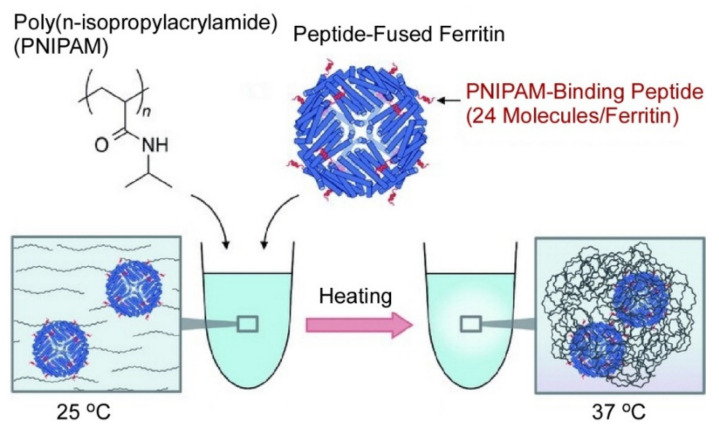
Complexes of ferritin and thermo-responsive polymer (poly(N-isopropylacrylamide)) formed with the aid of polymer-binding peptides. Reprinted with permission from Reference [167]. Copyright 2020 Chemical Society of Japan.

Lim, Serizawa, and co-workers developed complexes of a protein (ferritin) and thermo-responsive polymer (poly(N-isopropylacrylamide)) with the aid of polymer-binding peptides (Figure 22) [167]. Ferritin proteins obtained from the archaeon Archaeoglobus fulgidus have sufficiently high thermal stability. Cage structures were nanoarchitected through self-assembly of 24 polypeptides through protein shell formation around an insoluble ferric iron core. The formed cage had a hydrodynamic diameter of ca. 6 nm in its unloaded apo form and of ca. 12 nm in a cage architecture. Further modification with poly(N-isopropylacrylamide) on the cage was performed to complete the protein-polymer complexes mediated through recombinant peptide fusion. The phase transition of poly(N-isopropylacrylamide) upon gradual temperature increase fastened the complex nanoarchitectures. Release of ferritin from the complex was observed in 1 to 2 days at 37 °C. These complex formation processes are reversible, and structures including protein secondary structures are preserved during phase transitions. Passive releases of ferritin proteins from the complexes are sufficiently slow. The latter feature is advantageous for protein delivery with controlled systemic circulation times.

**Figure 23:**
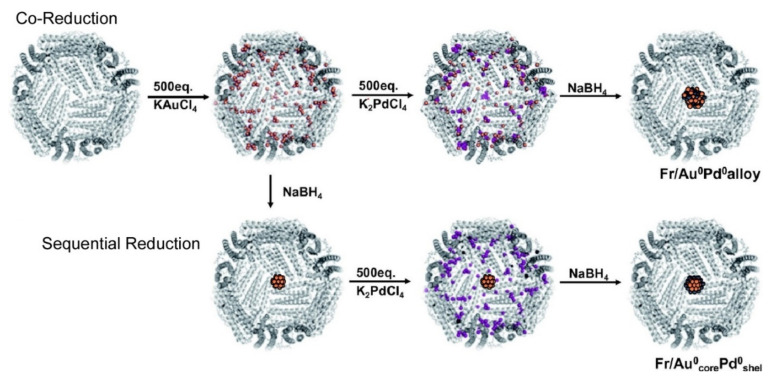
Nanospaces of the apo-ferritin forms used as reaction fields for metallic nanoarchitectonics such as preparation of alloy core-shell and bimetallic Au^0^/Pd^0^ nanoparticles. Reprinted with permission from Reference [168]. Copyright 2020 Chemical Society of Japan.

Cage nanoarchitectures of ferritin can be used for reaction media such as artificial metalloenzymes, as summarized in the recent review article by Watanabe et al. [168]. Incorporation of Pd^0^ nanoclusters, Pd^2+^ (η^3^-C_3_H_5_) complexes and Rh^1+^ (norbornadiene) complexes into the cage of apo-ferritin forms artificial metalloenzymes as reaction vessels for hydrogenation of olefins, Suzuki–Miyaura C–C coupling, and phenylacetylene polymerization, respectively. The nanospaces of the apo-ferritin forms can be used as reaction fields for metallic nanoarchitectonics such as preparation of alloy core-shell and bimetallic Au^0^/Pd^0^ nanoparticles (Figure 23). Au^0^/Pd^0^ alloy nanoparticles were synthesized upon co-reduction of Au^3+^ and Pd^2+^ ions immobilized in the apo-ferritin nanospace. In contrast, stepwise reduction of these two metal ions within the apo-ferritin gave core-shell Au^0^/Pd^0^ nanoparticles. Two step reductions with NaBH_4_ for Au^3+^ ion in the first step and Pd^2+^ ion in the second step resulted in formation of the core-shell Au^0^/Pd^0^ nanoparticles. The latter core-shell nanoparticles entrapped in the apo-ferritin nanospace exhibited much higher catalytic activities in olefin hydrogenation that those for the alloy nanoparticles within the ferritin, respectively. The importance of atom-level arrangement for catalytic functions of metallic clusters was demonstrated in metal nanoarchitectonics in protein cage spaces.

**Figure 24:**
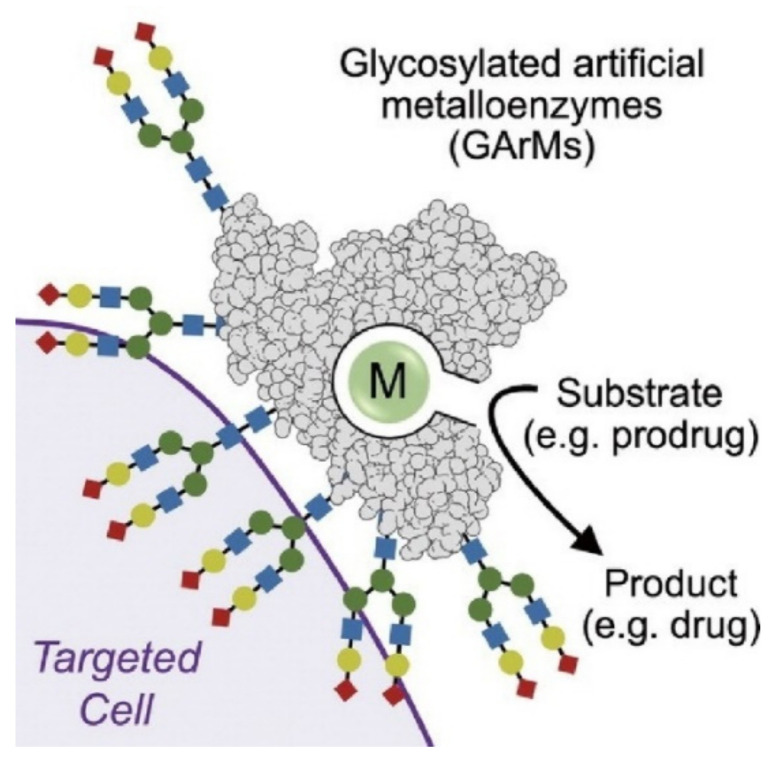
Glycosylated artificial metalloenzymes nanoarchitected based on in vivo synthetic chemistry; designed glucans are covalently introduced onto base protein. Reprinted with permission from Reference [169]. Copyright 2020 Chemical Society of Japan.

Glycosylated artificial metalloenzymes can be nanoarchitected on protein molecules (Figure 24) [169], as demonstrated in a recent review article by Tanaka and Vong. Based on in vivo synthetic chemistry, designed glucans are covalently introduced onto a base protein. Artificial metalloenzymes are further nanoarchitected on the basis of information from identification of glycan-dependent targeting and methodologies for biocatalyst functionalization. This biocompatible nanoarchitecture on proteins to produce glycosylated artificial metalloenzymes requires non-natural chemical reactions within living biological systems. Additional functions of molecular selectivity to artificial metalloenzymes [170] are useful in various application areas such as diagnostics, drug therapy, and pharmaceutical synthesis.

## 7. Bio-Related Nanoarchitectonics in Conjugation with Materials

**Figure 25:**
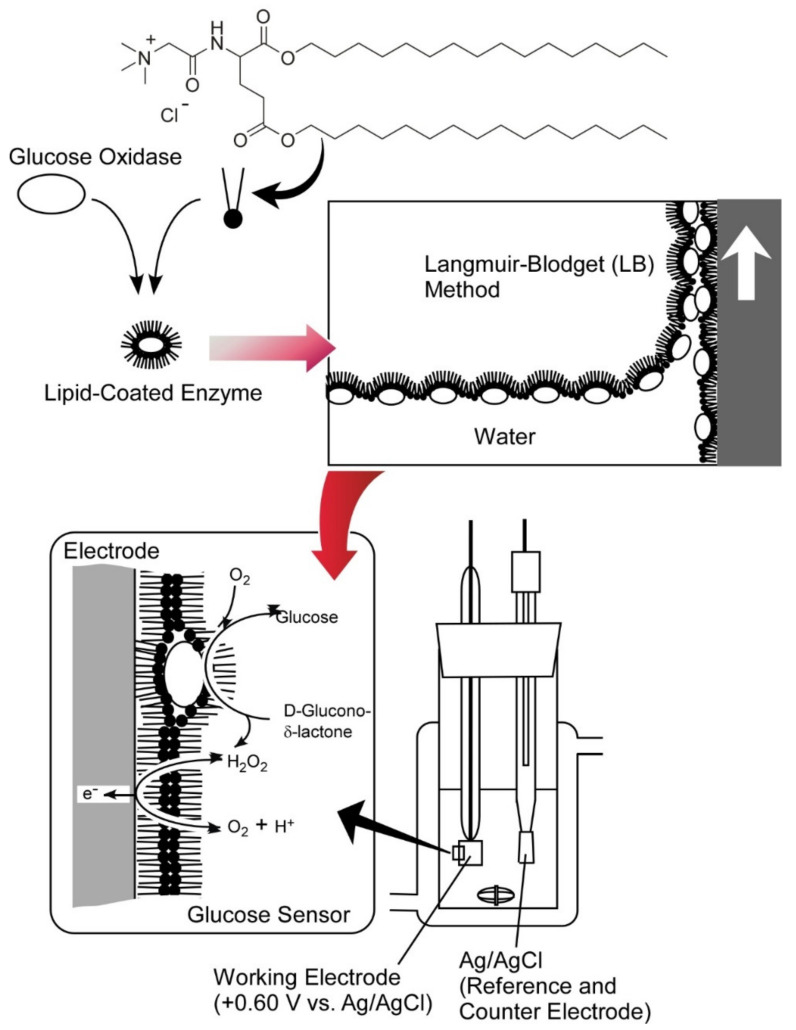
Monolayer transfer of the lipid-coated glucose oxidase onto electrode surface for preparation of sensitive sensors for glucose detection.

For further development of bio-functional systems, conjugation of bioactive components onto artificial devices and nanostructured materials is necessary [171,172]. One of the popular strategies in bio-device conjugation nanoarchitectonics is rational immobilization of bioactive components such as enzymes onto electrodes. Ultrathin film nanoarchitectonics of enzymes in electrodes are advantageous for quick-response sensor systems through barrier-less communication between enzyme reactions and electrochemical signal conversions at electrode sides. Although the Langmuir–Blodgett (LB) method is one of the most powerful techniques to immobilize functional components as monolayers and a few layer structures [173,174,175], exposure of enzymes at the air–water interface often causes serious denaturation of the target enzymes because of sufficiently high surface tension at the water surface. In order to avoid such disadvantageous features in the LB method, an LB process with lipid-coated enzymes was proposed. In Figure 25, glucose oxidase was coated with lipid molecules [176,177]. The lipid coated glucose oxidase molecules are soluble in organic solvent and thus can be spread at the air–water interface. Denaturation of glucose oxidase at the air–water interface was virtually avoided through the lipid-coated nanoarchitectonics. Monolayer transfer of the lipid-coated glucose oxidase onto the electrode surface was successfully performed without serious activity loss. The nanoarchitected enzyme-conjugated electrode systems can work as sensitive sensors for glucose detection through H_2_O_2_ production with enzymatic glucose oxidation to gluconolactone and subsequent electrochemical reaction of the resulting H_2_O_2_.

**Figure 26:**
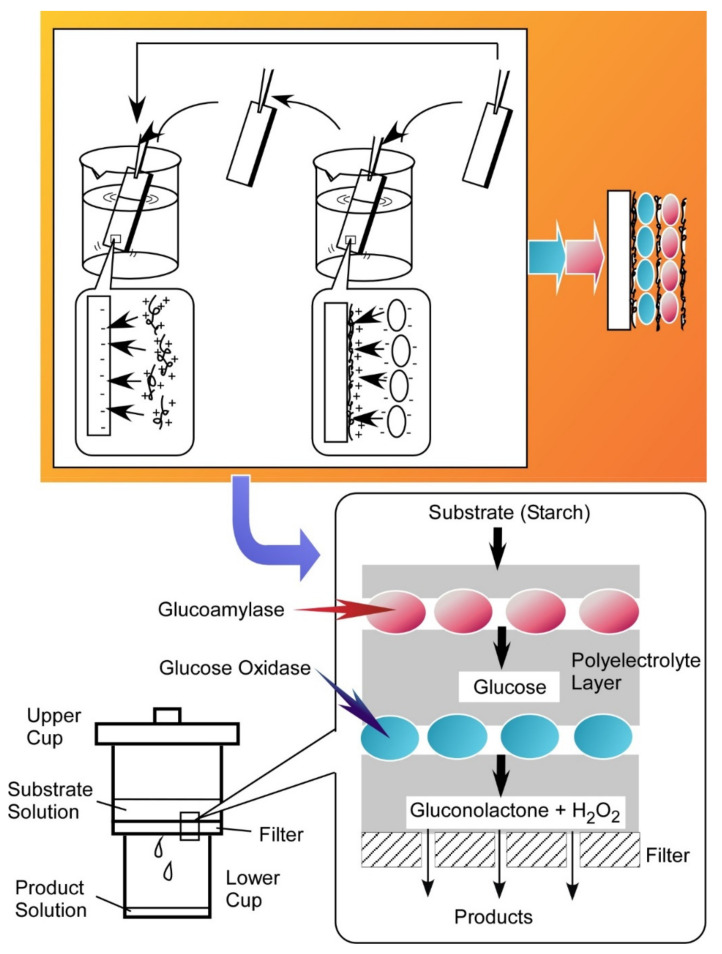
Multi-enzyme nanoreactors nanoarchitected on filter systems though sequential LbL assembly of glucoamylase and glucose oxidase with the aid of appropriate polyelectrolytes.

Another powerful method of thin-film nanoarchitectonics of bioactive components is LbL assembly [178,179,180]. Based on intermolecular interactions such as electrostatic interaction [181,182] and bio-specific recognition [183,184], various bioactive components can be immobilized as thin films with desired thicknesses and layer sequences. Glucose sensing systems can be similarly nanoarchitected by LbL assembly of glucose oxidase and its counterionic polyelectrolyte [185]. Multi-enzyme nanoreactors were nanoarchitected on filter systems though the sequential LbL assembly of glucoamylase and glucose oxidase with the aid of appropriate polyelectrolytes (Figure 26) [186]. In the latter case, sequential material conversions from starch to glucose and gluconolactone were successfully demonstrated if the layer order of the two kinds of enzymes matched well with the reaction sequence. In addition, co-assembly with polyelectrolyte drastically increased the stability of immobilized enzymes [187].

Matsuda et al. recently evaluated the direct electron-transfer reaction of cytochrome c that was immobilized onto a bare indium tin oxide electrode [188]. They used slab optical waveguide spectroscopy to perform in situ measurements of cytochrome c adsorbed on liquid–solid interfaces even with sub-monolayer coverage. The proposed measurement system enabled them to directly observe the direct electron-transfer reaction of the immobilized cytochrome c molecules through evaluation of spectral changes in the Soret band between the oxidized form at 408 nm and reduced form at 415 nm. In addition, evaluation with cyclic voltammograms could also yield more information on the direct electron-transfer activities of the cytochrome c immobilized on the indium tin oxide electrode.

**Figure 27:**
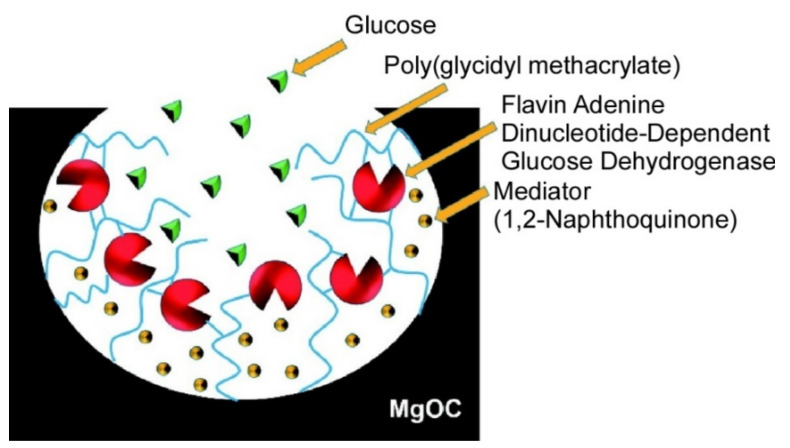
Stable immobilization of flavin adenine dinucleotide-dependent glucose dehydrogenase onto mesoporous carbon support through reaction with glycidyl groups attached to poly(glycidyl methacrylate). Reprinted with permission from Reference [194]. Copyright 2020 Chemical Society of Japan.

Immobilization of enzymes on nanostructured solid supports has been widely researched. Especially, various porous materials including nanoporous, mesoporous, and macroporous materials sometimes become attractive supports for enzyme immobilization due to their huge surface area, regular pore geometry, and good size matching with the enzymes [189,190]. Fujiwara et al. demonstrated the immobilization of esterase molecules obtained from Acinetobacter calcoaceticus F46 onto macroporous silica microcapsules [191]. Reaction rates for the hydrolysis reaction of 3,4-dihydrocoumarin catalyzed by the esterase immobilized on the macroporous silica microcapsules were actually greater than those for the esterase on mesoporous silica support. The larger pore size of the macroporous supports (ca. 500 nm) facilitated access of reacting species to the esterase. Even though the esterase was immobilized onto pores inside, it exhibited activities comparable with those observed for non-immobilized esterase. Porous carbon materials are also used as supports for immobilization of bioactive materials [192,193]. For example, stable immobilization of flavin adenine dinucleotide-dependent glucose dehydrogenase onto a mesoporous carbon support through reaction with glycidyl groups attached to poly(glycidyl methacrylate) was demonstrated by Shitanda et al. [194]. As shown in Figure 27, poly(glycidyl methacrylate) was grafted onto the surface of MgO-templated carbon in the first step, followed by introduction of 1,2-naphthoquinone as a mediator. Molecules of the enzyme, flavin adenine dinucleotide-dependent glucose dehydrogenase, were finally immobilized on MgO-templated carbon through linking with epoxy groups. Catalytic current upon glucose oxidation by the enzyme could be transmitted to electrodes via 1,2-naphthoquinone mediator. These immobilization processes lead to stable operation of the modified electrodes with long-term storage and continuous performance.

From an opposite perspective, biomaterials can be used for nanoarchitectonics of other materials, as seen in biomass-derived nanomaterials preparation [195,196]. Xu, Wang, Hossain, and co-workers demonstrated preparation of porous carbon materials from lignocellulose as a biomass source and application of synthesized carbon for high capacitive deionization [197]. Synthetic procedures are just based on direct carbonization of lignocellulose. The resulting porous carbon materials exhibited high salt adsorption capacity and good cycling stability. From a practical application perspective, various advantages such as environmental friendliness, high production yields, and cost-effectiveness are included. These nanoarchitectonics approaches are rather simple but fit perfectly with sustainable and green technologies.

## 8. Summary and Future Perspectives

Production of functional materials by nanoarchitectonics is somewhat similar to the creation of functional systems in biological systems. Therefore, it can be said that the goal of nanoarchitectonics is to create highly functional systems similar to those found in biological systems. In this review, several examples on nanoarchitectonics processes with biomolecules and biomimetic molecules were briefly explained according to classifications of components. Bottom-level nanoarchitectonics for biomimetic and biological functional materials can be based on molecular conversions to create complicated functional molecules from rather simple molecular components. While well-established organic synthesis has crucial roles in bottom-level nanoarchitectonics, nanotechnological tools were recently introduced for chemical syntheses. Highly site-specific molecular conversions are possible using a probe tip for scanning microscopic spectroscopy.

Most of the important functional systems in biology are created through the self-assembly/self-organization of component biomolecules. Therefore, self-assembly nanoarchitectonics has been widely researched using various biomolecules and biomimetic molecules. Nanoarchitectonics with nucleic acids (mainly DNA and RNA) are performed on the basis of strict base pairing (molecular recognition) upon complemental hydrogen bonding. Self-assembling motifs are not limited to standard double helix formations from two strands. Non-double strand helix and branched assemblies from more than two strands can be formed, where strict base pairing is, however, retained as the principal mechanism. With this high capability of structure formation, peptide units have been used for active components in self-assembly nanoarchitectonics. Even short peptides such as dipeptides and tripeptides can form regular nanoarchitectures, and more advanced assembled structures also can be nanoarchitected with longer peptides with defined designs. Research has also been performed on the development of protein mimicry molecules and functional modulation and assemblies of proteins themselves. Enzymes are often nanoarchitected onto devices such as electrodes and/or nanoporous materials. Conjugation with artificial non-biological structures is an effective way to fabricate functional systems. Although related examples are not mentioned in detail in this review, other biocomponents such as oligosaccharides can be used for production of functional material systems [198,199,200].

Although various successful examples for functional systems have been created through nanoarchitectonics using biomimetic and biomimetic molecular components, the preparation of bio-like functional material systems with numerous molecular components with complicated hierarchical architectures is actually a tough target. Nature has successfully created such sophisticated functional systems over billions of years. In order to produce bio-comparable functional materials with nanoarchitectonics strategies within a few decades, some additional elements will be necessary. One solution would be the use of high functional living systems as nanoarchitectonics components instead of constructing functional systems totally from molecular bottoms. Utilizing nanoarchitectonics to integrate living cells with sophisticated functions into artificially designed architectures would be a promising approach to create bio-like functional systems. Methodologies for living cell assemblies are actually being developed, as observed with cell sheet technologies [201,202] and the LbL assembly of living cells [203,204]. In addition, living cell cultures at the liquid–liquid interface between the aqueous phase and fluorocarbon phase are possible, and consequently, the LB transfer of living cells onto a solid surface has actually been demonstrated [205,206,207]. The orientation, arrangement, and differentiation of living cells are regulated at various interfaces [208,209,210]. By using these nanoarchitectonics techniques, living cells can be organized into functional architectures together with nanoarchitectonics of smaller biomolecules and non-biomolecules. Because of the high functional capabilities of component cells, organized systems with much higher functions can be created with this nanoarchitectonics strategy. Another element for the creation of bio-like functional systems is the use of artificial intelligence. In many biological functional systems, multiple types of components make functional contributions and work together in complicated relationships. This complexity is essential for high functions in biological systems. However, the design of whole systems of this kind is not so easy with traditional materials and design methods. The use of machine learning is proposed for the optimization of material production and understanding of related phenomena [211,212,213]. The use of material informatics for nanoarchitectonics on functional porous materials also has been proposed [214]. Rapidly developing methods based on artificial intelligence would be used for the creation of bio-like highly functional material systems within a short period, whereas real biological systems evolved over billions of years.
